# Supplementary material for: Biosecurity Uptake and Perceived Risk of Avian Influenza Among People in Contact With Birds
Source: Zoonoses Public Health. 2026 Jan 6;73(2):152–63. doi: 10.1111/zph.70034 (PMC12865334; doi:10.1111/zph.70034)
Supplement: Supplementary file 1 — FIGURE S1: Frequency counts of different biosecurity measures in each biosecurity group. (a) Use of at least one PPE measure for the face or body, (b) use of at least one footwear related PPE measure and (c) use of at least one food safety measure. Use of biosecurity measures were recorded using radio buttons that respondents ‘checked’ for yes. We assumed responses with all measures ‘unchecked’ indicated a skipped question and were therefore coded as missing. FIGURE S2: Frequency counts of risk perception categories for risk of avian influenza to (a) respondents' health and (b) risk to the health of their birds. TABLE S1: Frequency of respondents who used at least one biosecurity measure from each biosecurity group. [file ZPH-73-152-s001.docx]

# **Biosecurity uptake and perceived risk of avian influenza among people in contact with birds**

Harry Whitlow^1^*, Suzanne Gokool^1^, Genevieve Clapp^1^, Irene Bueno^2^, Mariam Logunleko^2^,
Peter Moore^2^, Sarah Masterton^3^, Jo Taylor-Egbeyemi^3^, Ian Brown^4^, Riinu Pae^3^, Louise E Smith^3^, Ellen Brooks-Pollock^1, 5^, Amy C Thomas^1^**

1: Population Health Sciences, Bristol Medical School, University of Bristol, Bristol, UK

2: Bristol Veterinary School, University of Bristol, Langford, UK

3: Behavioural Science and Insights Unit, UK Health Security Agency, London, UK

4: The Pirbright Institute, Pirbright, England, UK

5: UK NIHR Health Protection Research Unit in Behavioural Science and Evaluation

*Corresponding author 1: [ja23401@bristol.ac.uk](mailto:ja23401@bristol.ac.uk)

**Corresponding author 2: [amyc.thomas@bristol.ac.uk](mailto:amyc.thomas@bristol.ac.uk)

**
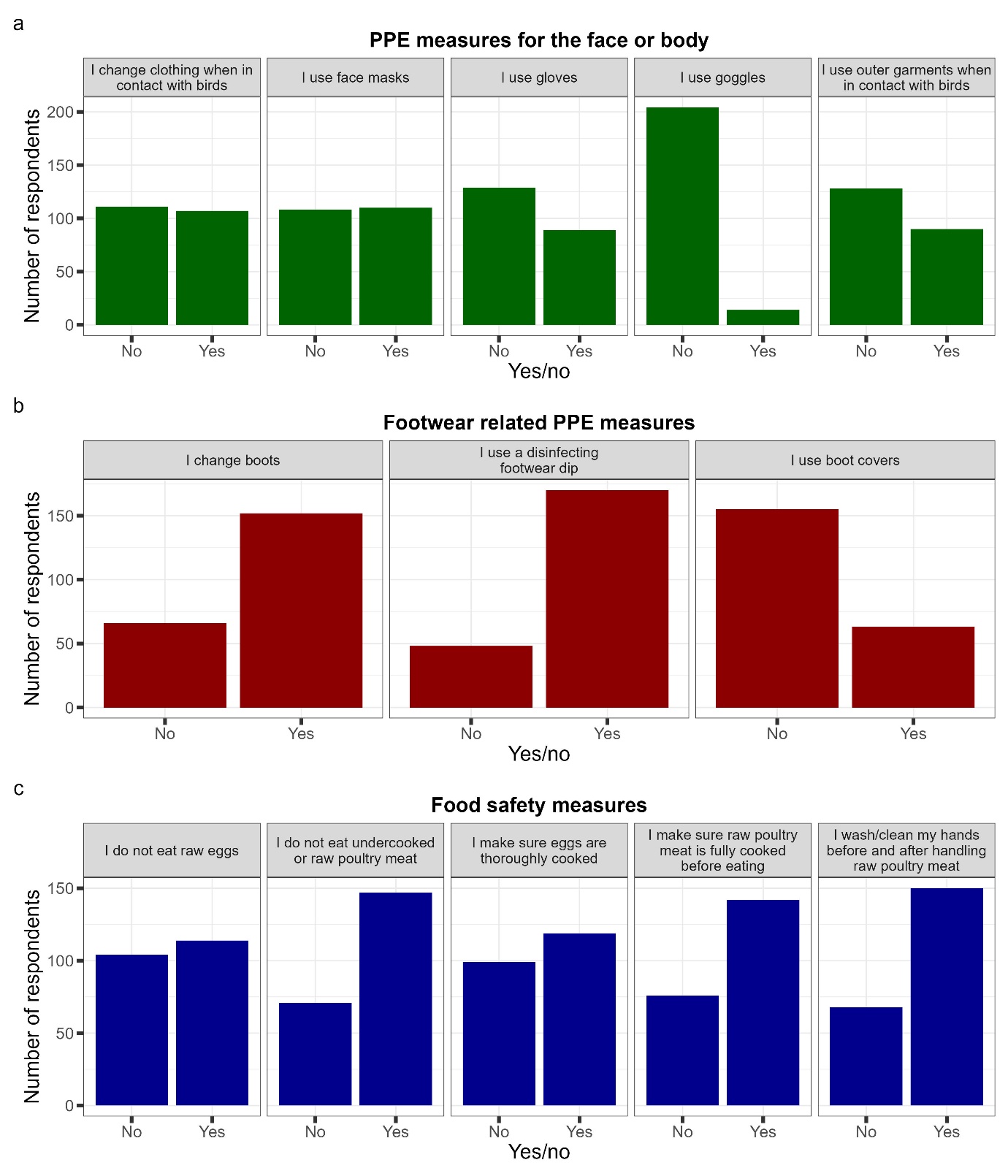
Figure S1.** Frequency counts of different biosecurity measures in each biosecurity group. (a) Use of at least one PPE measure for the face or body, (b) use of at least one footwear related PPE measure and (c) use of at least one food safety measure. Use of biosecurity measures were recorded using radio buttons that respondents “checked” for yes. We assumed responses with all measures “unchecked” indicated a skipped question and were therefore coded as missing.

**
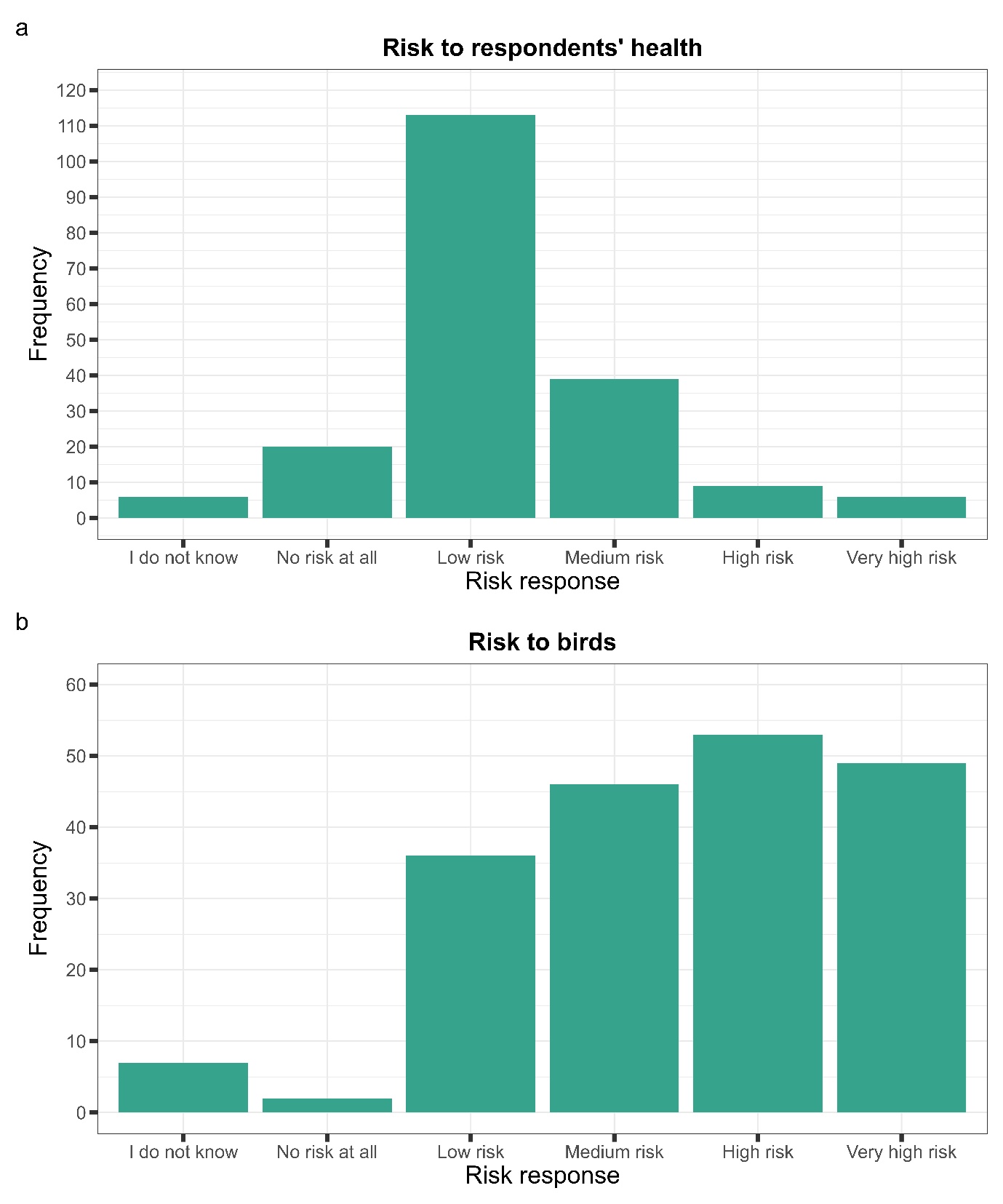
Figure S2.** Frequency counts of risk perception categories for risk of avian influenza to (a) respondents’ health and (b) risk to the health of their birds.

**Table S1.** Frequency of respondents who used at least one biosecurity measure from each biosecurity group.

| **Biosecurity measure group** | **Number of respondents using at least one measure in the group (n=217):** |
| --- | --- |
| PPE measures for the face or body | 181 (83.4%) |
| Footwear related measures | 187 (86.2%) |
| Food safety measures | 180 (82.9%) |
